# Apricot Melanoidins Prevent Oxidative Endothelial Cell Death by Counteracting Mitochondrial Oxidation and Membrane Depolarization

**DOI:** 10.1371/journal.pone.0048817

**Published:** 2012-11-08

**Authors:** Annalisa Cossu, Anna Maria Posadino, Roberta Giordo, Costanza Emanueli, Anna Maria Sanguinetti, Amalia Piscopo, Marco Poiana, Giampiero Capobianco, Antonio Piga, Gianfranco Pintus

**Affiliations:** 1 Department of Biomedical Sciences, University of Sassari, Sassari, Italy; 2 Centre of Excellence for Biotechnology Development and Biodiversity Research, University of Sassari, Sassari, Italy; 3 Department of Agriculture, University of Sassari, Sassari, Italy; 4 Department of Biotechnologies for Agricultural Food and Environmental Monitoring, Mediterranean University of Reggio Calabria, Feo di Vito, Reggio Calabria, Italy; 5 Laboratory of Vascular Pathology and Regeneration, Regenerative Medicine Section, School of Clinical Sciences, Bristol Heart Institute, University of Bristol, Bristol, United Kingdom; 6 Gynecologic and Obstetric Clinic, University of Sassari, Sassari, Italy; University of Pecs Medical School, Hungary

## Abstract

The cardiovascular benefits associated with diets rich in fruit and vegetables are thought to be due to phytochemicals contained in fresh plant material. However, whether processed plant foods provide the same benefits as unprocessed ones is an open question. Melanoidins from heat-processed apricots were isolated and their presence confirmed by colorimetric analysis and browning index. Oxidative injury of endothelial cells (ECs) is the key step for the onset and progression of cardiovascular diseases (CVD), therefore the potential protective effect of apricot melanoidins on hydrogen peroxide-induced oxidative mitochondrial damage and cell death was explored in human ECs. The redox state of cytoplasmic and mitochondrial compartments was detected by using the redox-sensitive, fluorescent protein (roGFP), while the mitochondrial membrane potential (MMP) was assessed with the fluorescent dye, JC-1. ECs exposure to hydrogen peroxide, dose-dependently induced mitochondrial and cytoplasmic oxidation. Additionally detected hydrogen peroxide-induced phenomena were MMP dissipation and ECs death. Pretreatment of ECs with apricot melanoidins, significantly counteracted and ultimately abolished hydrogen peroxide-induced intracellular oxidation, mitochondrial depolarization and cell death. In this regard, our current results clearly indicate that melanoidins derived from heat-processed apricots, protect human ECs against oxidative stress.

## Introduction

An inverse correlation between a diet rich in plant foods and the occurrence of cardiovascular diseases (CVD) has been reported in several epidemiological studies [1]. The vasculoprotective effect associated to fruit and vegetable consumption is thought to be due to fresh plant-contained phytochemicals, including antioxidant substances such as phenolic compounds, carotenoids and vitamins [1]. However, a remarkable amount of the food intake in the human diet comes from processed foodstuffs, and whether processed plant-foods provide less benefit than unprocessed ones remains an area of inquiry.

One of the main food unit operations is based on thermal treatments. Heat-based food transformations often result in non-enzymatic browning (NEB), which occurs through sugars thermal degradation, or, under acidic conditions, by the Maillard Reaction (MR) between sugars and organic acids [2]. During the last stage of the NEB reaction high molecular-weight heterogeneous polymers called melanoidins are formed [2]. Melanoidins are widely distributed in processed foods and may have various *in vitro* functional properties, including antioxidant [3], [4], antihypertensive [5] and metal-binding activities [6]. The antioxidant activity of melanoidins is of particular interest since it can influence the oxidative and shelf life of several foods during storage [7], [8]. In line with the observed antioxidant activity, some biological effects, including cell protection against oxidative damage, have been reported for coffee, biscuit and prune melanoidins [9]–[11]. However, because of the huge complexity of both reactions and products during their chemical pathway of formation, only partial structures of melanoidins have been elucidated so far [2]. Thus it is very difficult to address a specific health effect to a distinctive melanoidin chemical structure; therefore a deep and accurate exploration is needed for melanoidins derived from different foods.

Apricot fruits are considered as a rich source of phytochemicals, which are mainly polyphenols and carotenoids [12], [13]. Phenolic compounds, in particular, by acting as antioxidants, are thought to provide various *in vivo* health benefits including hepato- and cardio-protective effects [14], [15]. The antioxidant properties of polyphenols in apricots have been studied in relation to ripening, cultivar and puree preparation [13], [16], [17], and contrasting results about the antioxidant activity of fresh apricot fruits have been often reported [18], [19]. However, 40–45% of the total world production of apricots is processed, mainly by drying and thermal treatment [20]. Similar to our previous finding on prunes [3], we found that drying apricots at high processing temperatures resulted in a significant increase of antioxidant activity, even though the phenol content was significantly reduced [21]. We hypothesized that the increased in antioxidant activity observed in the dried apricots might have been due to the formation of NEB products (NEBPs), after drying (e.g. melanoidins). Thus, as reported for prunes [3], melanoidins appear to be the prevailing contributors to the maintained antioxidant activity of dried apricot *in vitro*. In this regard, although the antioxidant properties of melanoidins have been studied *in vitro* for several years their potential antioxidant effects on human biological systems remains largely unknown.

The finding that oxidative stress is a common feature in many aspects of CVD pathogenesis [22], suggests that its counteraction with antioxidants may prevent disease occurrence or ameliorate a patient’s pathological condition. For this reason a great deal of attention is now focusing on naturally occurring antioxidants as potential candidates for CVD prevention and/or treatment. Endothelial cells (ECs) play a crucial role in the integration and modulation of signals within the vascular wall [23] and perturbation of such homeostasis by oxidative damage is the trigger for the development of CVD [24]. We have previous reported that melanoidins obtained from prunes protect human ECs from hydrogen peroxide-induced oxidative stress and cell death [11], but whether such kind of cellular protection is also provided by melanoidins isolated from apricots is completely unknown. Indeed, chemical characteristics, both quantitative and analytical, of compounds that participate in melanoidins formation in prunes and apricots are known to differ and thus different melanoidins may originate from their processing [21], [25], [26].

Hence, the present work was undertaken with the intent to investigate whether the food melanoidins isolated from dried apricots might protect human ECs against H_2_O_2_-induced oxidative stress and cell damage.

## Results

### Thermal Treatment Increases NEB in Processed Apricots

**Figure 1:**
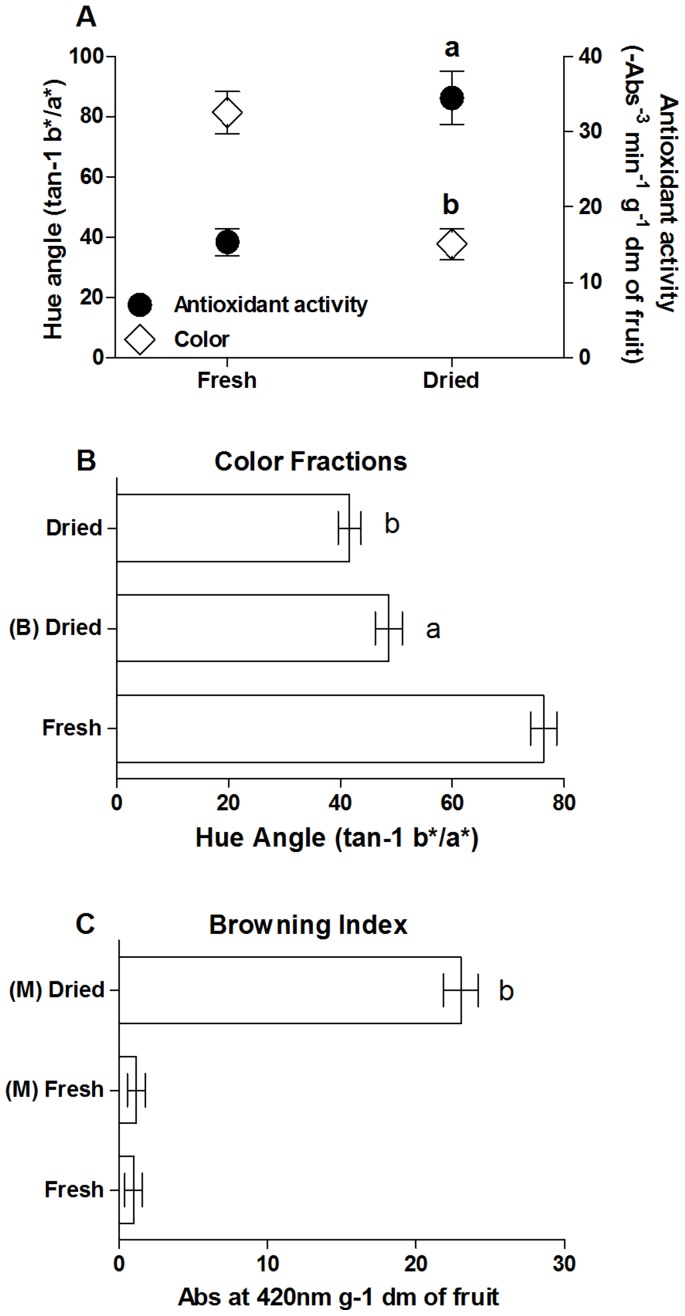
Changes in chemical parameters elicited by fruit processing. (A) Changes in antioxidant activity and color expressed as hue variation (tan - 1 b*/a*). (B) Changes in color expressed as hue variation (tan - 1 b*/a*). (C) Changes in color expressed as browning index (Abs at 420 nm g^−1^ dm of fruit). Fresh, fresh fruits; M, melanoidins; B, blanching. Data are the mean ± SE from four or five measurements. (A–C) a; b, significantly different from the fresh sample.

A significant increase in antioxidant capacity was elicited by fruit drying, which strongly correlated with the paralleled increase of color found in the same polyphenolic extract (Fig. 1A). Colour was indeed dramatically changed by the drying process with a significant reduction of tonality (Fig. 1B). In particular, the blanching of sample resulted in a significantly lower reduction of the drying-induced variation of color, which is expressed as a hue angle in the Hunter scale [27], [28]. In fact, a more pronounced shift to a redder and deeper zone in the Hunter scale is evident in the dried, control fruits, with respect to the blanched ones, which might depend on the enzymatic browing contribution [29]. However, in blanched fruits where no enzymatic browning is present [30], the observed color variation can be explained only by the formation of NEB during the drying process (Fig. 1B). Browing index analysis of the melanoidin fractionsisolated from fresh and processed apricots indicated that NEBPs were present only in dried fruits, thus confirming melanoidins presence in the extract obtained from the processed samples (Fig. 1C). Among all the melanoidins fractions, Fraction I showed the highest amount of NEBPs as measured at 420 nm (data not shown), and was therefore chosen to be tested for its antioxidant activity on cells exposed to oxidative stress.

### Hydrogen Peroxide Induces Mitochondrial Damage and Cell Death

**Figure 2:**
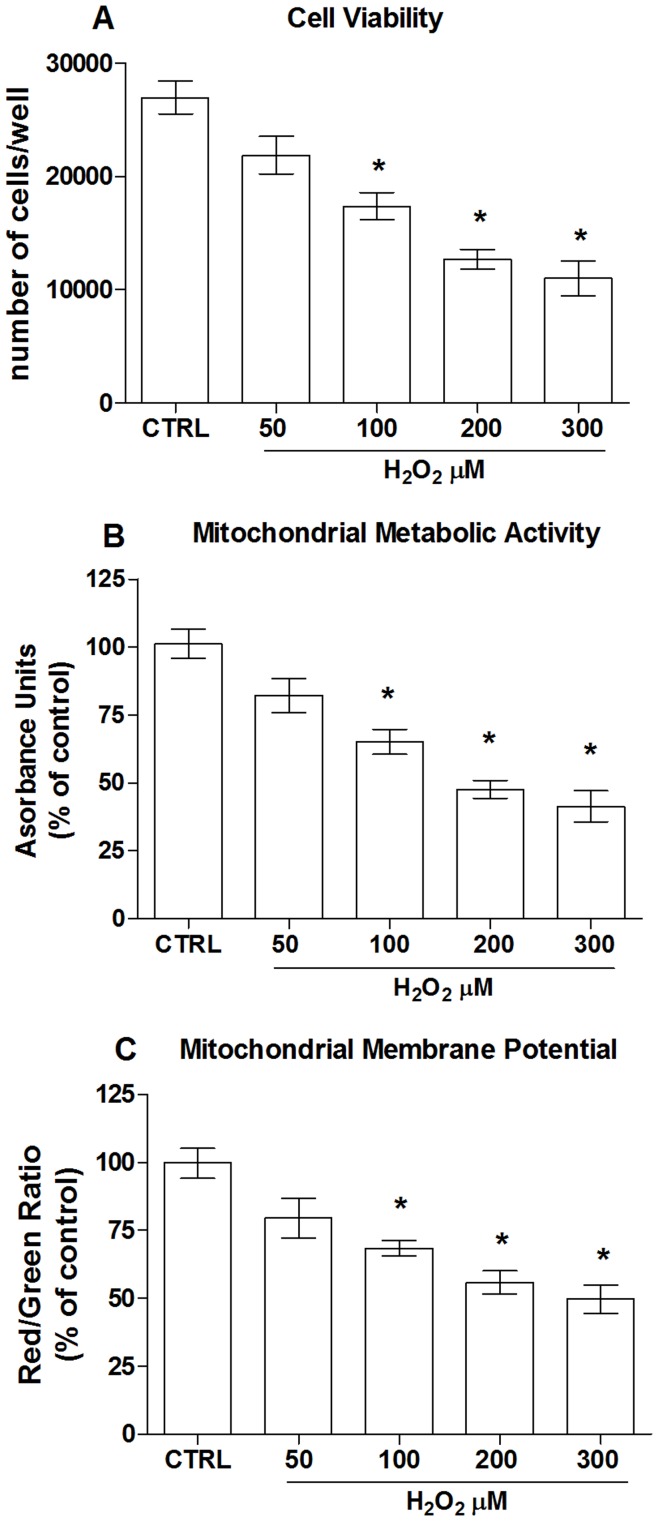
Hydrogen peroxide induces mitochondrial damage and cell death. Dose-dependent effect of hydrogen peroxide (H_2_O_2_) on (A) cell viability, (B) mitochondrial metabolic activity and (C) mitochondrial membrane potential. Data are the mean ± SE of four experiments. (A–C) *, significantly different from the control.

In order to mimic oxidative damage we investigated the effect of different doses of H_2_O_2_ on ECs death measured as cell viability and mitochondrial metabolic activity (MMA). As expected, 2 hr-treatment of ECs with H_2_O_2_ resulted in a dose-dependent reduction of cell survival as indicated by the significant decrease in the number of viable cells in comparison with the untreated control group (Fig. 2A). Consistently a significant decline of the MMA was concomitantly observed with the increasing doses of H_2_O_2_ (Fig. 2B). The oxidant-induced cell impairment was indeed associated with a superimposable loss of mitochondrial membrane potential (MMP), clearly indicating mitochondria implication in the hydrogen peroxide-induced cellular damage (Fig. 2B). Based on these experiments, around 50% of H_2_O_2_-induced mitochondrial impairment and cell death was observed at 100 µM of H_2_O_2_, and therefore this concentration was used to mimic oxidative stress in the next experiments.

### Apricot Melanoidins Protect Endothelial Cells from Hydrogen Peroxide-induced Mitochondrial Damage and Cell Death

**Figure 3:**
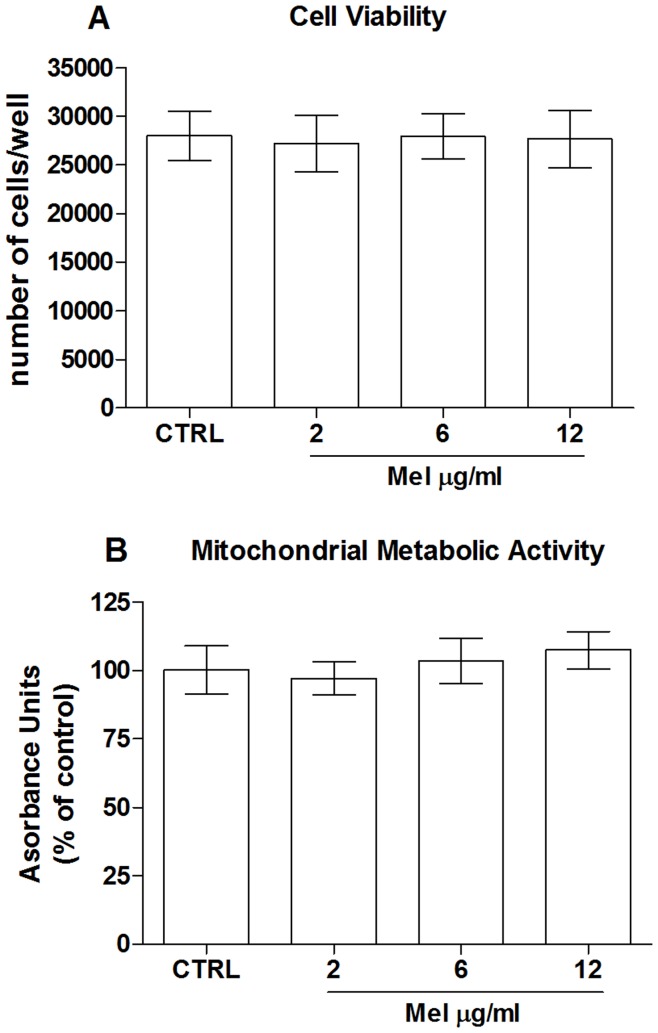
Apricots melanoidins are not toxic for endothelial cells. Effect of different concentrations of melanoidins on (A) cell viability and (B) mitochondrial metabolic activity. Data are the mean ± SE of four experiments.

**Figure 4:**
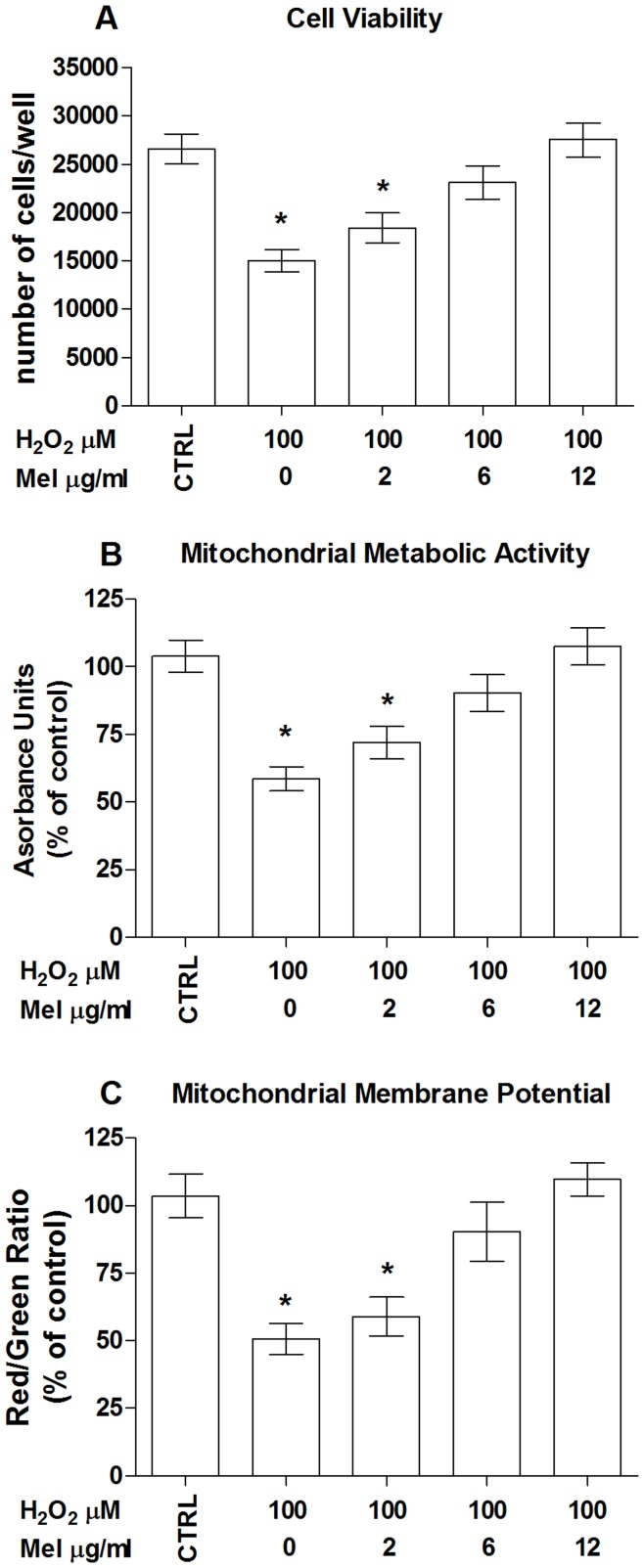
Melanoidins protect endothelial cells from hydrogen peroxide-induced mitochondrial damage and cell death. Dose-dependent effect of melanoidins on hydrogen peroxide (H_2_O_2_)-induced (A) cell death, (B) mitochondrial metabolic activity and (C) mitochondrial membrane potential. Data are the mean ± SE of four experiments. (A–C) *, significantly different from the control.

We first tested apricot melanoidins for potential toxicity in our human endothelial cells model. Based on previous observations concerning melanoidins from other sources of food such as coffee [9] and biscuit [10], we tested apricot-melanoidin at the concentrations of 2, 6 and 12 µg/ml at 48 hrs of stimulation, then melanoidin-treated cells were compared to untreated ones for both cell viability and MMA. Results shown in figure 3, which depict respectively lactate dehydrogenase (LDH) release and 3-(4,5-dimethythiazol2-yl)-2,5-diphenyl tetrazolium bromide (MTT) reduction, clearly indicate that apricot melanoidins did not produce toxic effects for the cells under the employed experimental conditions. We have previous demonstrated that pretreatment of human ECs with prune melanoidins [11] exerts remarkable protection against oxidative-induced cell death. However, melanoidins from different foods may have different structures and activities [2]. Indeed, the exact sequence of reactions from which melanoidins originated, as well as their chemical structures, in different food remain largely unknown [2]. We therefore asked whether, as with those isolated from prunes, melanoidins isolated from apricots could exert a protective effect on the observed H_2_O_2_-induced cell impairment. To this end, cells were treated with apricot melanoidins for 6 hrs and then H_2_O_2_ was added during the last 2 hrs of incubation to induce oxidative stress. As shown in figure 4A melanoidins pretreatment was able to dose-dependently counteract the decrease in cell viability induced by 100 µM H_2_O_2_. Failure of oxidant in eliciting MMP and MMA impairment in melanoidin-pretreated cells_,_ strongly indicates a protective effect of these compounds against oxidative stress and mitochondrial-mediated ECs death (Fig. 4B–C).

### Hydrogen Peroxide Induces Oxidation of Both Cytosolic and Mitochondrial Compartments

**Figure 5:**
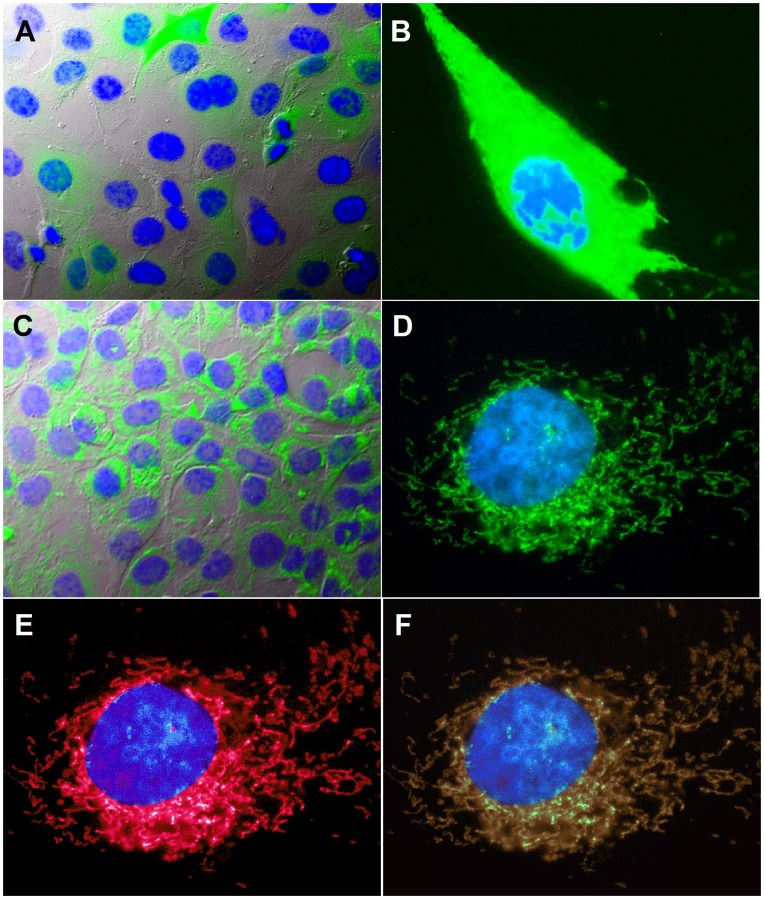
ECV304 cells lines constitutively expressing the cytoplasmic (cyto-roGFP) and mitochondrial (mito-roGFP) form of roGFP. Cells were grown in glass chamber slides at concentrations to allow 50–70% confluence in 24 hrs. On the day of experiments, cells were washed with PBS three times, counterstained with the mitochondrial marker MitoTracker Red and the nuclear marker ***Hoechst***, fixed with 4% paraformaldehyde and mounted for fluorescence microscopy visualization. Images (A) and (C) depict respectively merged photos of ECV304 cells expressing the cyto-and mito-*roGFP* (green) protein, ***Hoechst*** staining (blue) and bright-field (40X, NA = 1.00). Images (B) and (D) depict respectively merged photos of ECV304 cells expressing the cyto-and mito-*roGFP* (green) protein, counterstained with ***Hoechst*** (blue) (100X, NA = 1.35)). The figure 5F, depicts the merged photo of ECV304 cells expressing the mito-roGFP protein (D) and ECV304 cells stained with the mitochondrial marker MitoTracker Red (E). (100X, NA = 1,35)).

To further investigate the molecular mechanisms underpinning apricot melanoidins protection, we use two human EC lines constitutionally expressing the redox-sensing green fluorescent protein (roGFP) in both the cytosolic (cyto-roGFP) and mitochondrial (mito-roGFP) compartment (Fig. 5A–E). In particular, the fluorescence photo shown in figure 5B corresponds to ECV304 cells expressing the cyto-roGFP, while the fluorescence image shown in figure 5D represents ECV304 cells expressing mito-roGFP. Expression of the mito-roGFP in the mitochondrial compartment is confirmed by the image shown in figure 5F, which depicts the merged photo of ECV304 cells expressing the mito-roGFP (D) and ECV304 cells stained with the mitochondrial marker MitoTracker Red (E).

**Figure 6:**
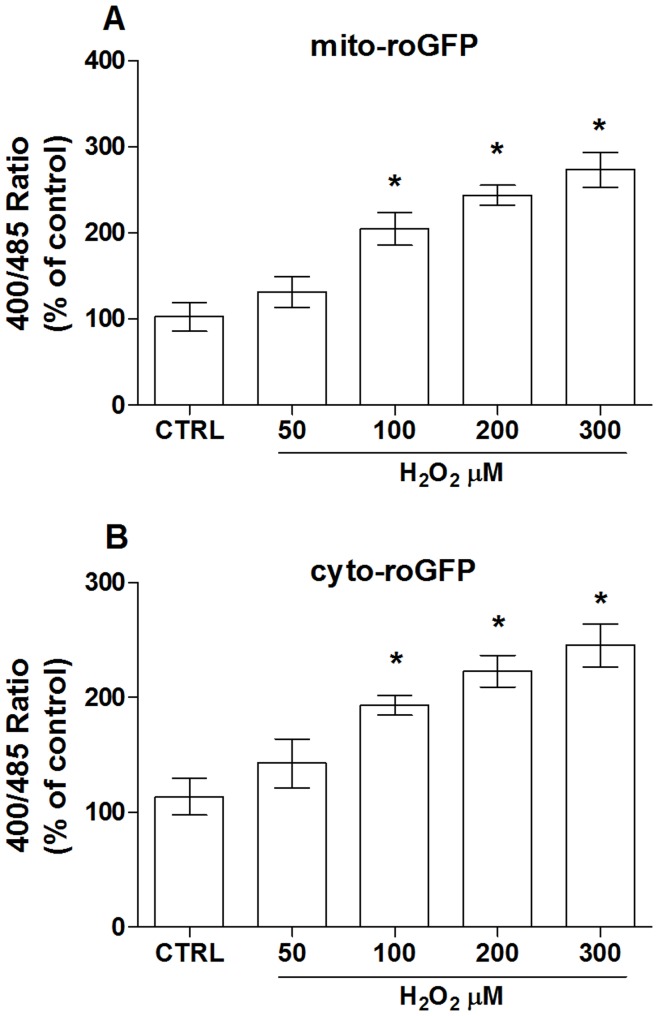
Hydrogen peroxide induces oxidation of both cytosolic and mitochondrial compartments. Dose-dependent effect of hydrogen peroxide (H_2_O_2_) on mitochondrial (mito-roGFP) and cytoplasmic (cyto-roGFP) ro-GFP oxidation. Data are the mean ± SE of four experiments. (A–B) *, significantly different from the control.

While the two images displayed in panels A and C of figure 5, depict the merged photos of ECV304 cells expressing the cytoplasmic (A) and the mitochondrial (C) form of the roGFP (green), along with the bright-field (40X magnification). From A–F, the nuclei are stained with hoechst (blue). These two cell lines allowed us to specifically follow potential changes of the cytosolic and mitochondrial redox state during our experimentation. As shown in Figure 6A–B, the treatment of roGFP expressing cells with H_2_O_2,_ concentration-dependently shifted the intracellular redox status *toward a more oxidative condition* in both the mitochondrial and cytosolic compartment, indicating that under our experimental conditions roGFP has a significant dynamic range and responds linearly to increasing doses of a well-known oxidant.

### Apricot Melanoidins Protect Cytosolic and Mitochondrial Compartments from Hydrogen Peroxide-induced Oxidative Redox Changes

**Figure 7:**
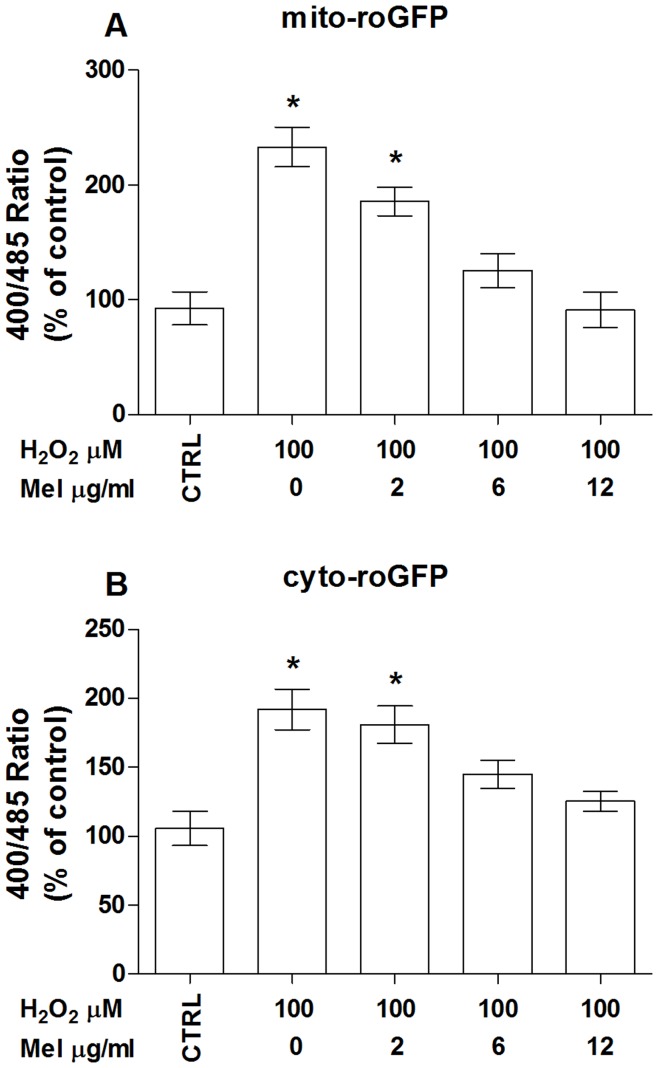
Melanoidins protect human endothelial cells from hydrogen peroxide-induced intracellular oxidative stress. Dose-dependent effect of melanoidins on H_2_O_2_-induced cytoplasmic (cyto-roGFP) and mitochondrial (mito-roGFP) roGFP oxidation. Data are the mean ± SE of four experiments. (A–B) *, significantly different from the control.

**Figure 8:**
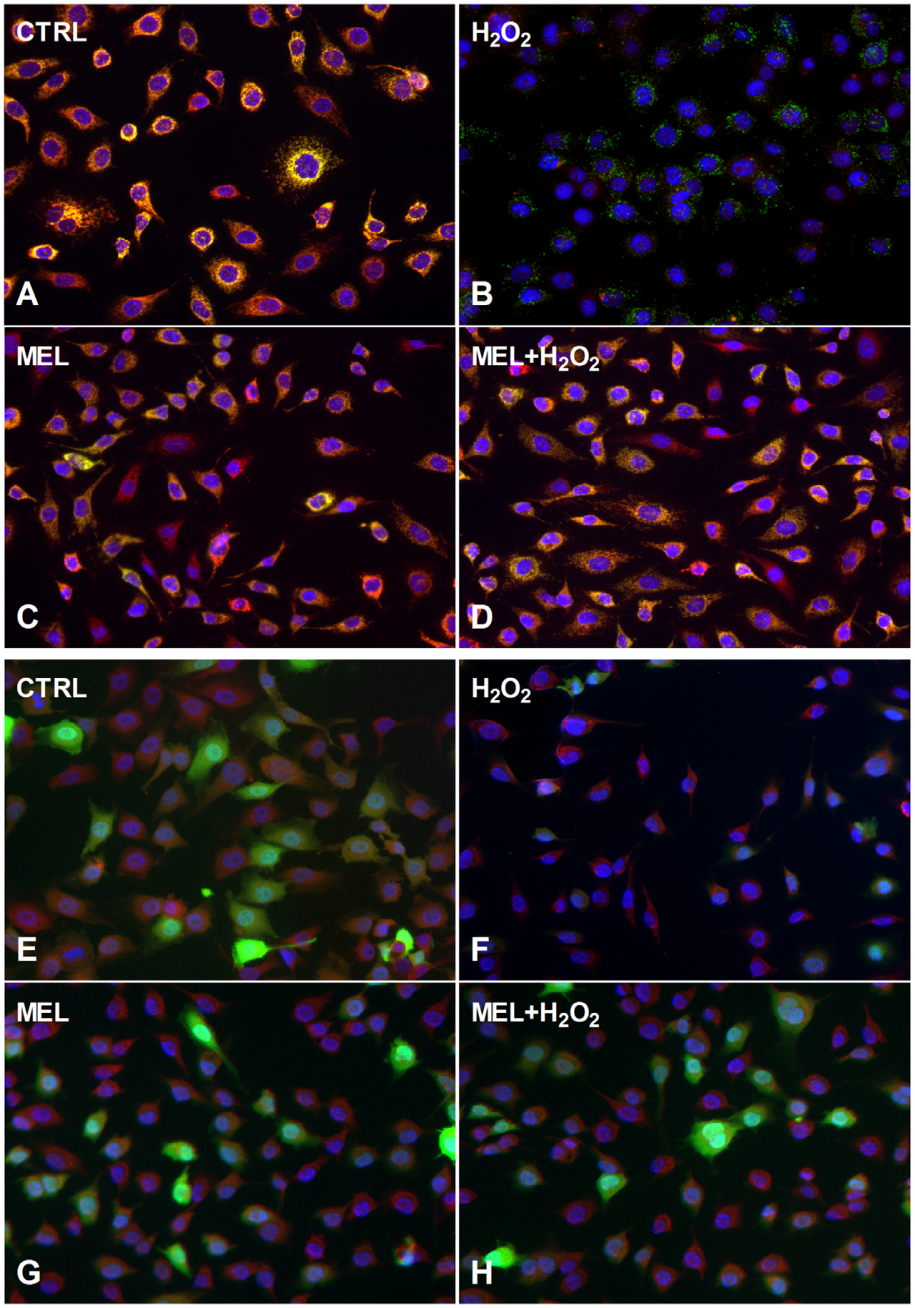
Melanoidins protect human endothelial cells from hydrogen peroxide-induced intracellular oxidative stress. Cells were grown in glass chamber slides at concentrations to allow 50–70% confluence in 24 hrs. On the day of experiments, cells were treated with apricot melanoidins for 6 hrs and then H_2_O_2_ was added during the last 2 hrs of incubation to induce oxidative stress. Then cells were washed with PBS three times, counterstained with the mitochondrial marker MitoTracker Red and the nuclear marker Hoechst, fixed with 4% paraformaldehyde and mounted for fluorescence microscopy visualization. Images (A–D) depict merged photos of ECV304 cells expressing the mito-*roGFP* (green) protein, counterstained with both MitoTraker Red and Hoechst. Images (E–H) depict merged photos of ECV304 cells expressing the cyto-*roGFP* (green) protein, counterstained with both MitoTraker Red and Hoechst. From A–H, magnification was 20X and NA 0,70).

We next wanted to determine whether the cellular protection elicited by melanoidins was due to the counteraction of H_2_O_2_-induced intracellular oxidation. To this end, roGFP expressing cells were treated with melanoidins for 6 hrs and 100 µM of H_2_O_2_ was added during the last 2 hrs of incubation. At the end of the experiment both mito- and cyto-roGFP fluorescence were recorded. Data shown in figure 7 A–B indicate that melanoidins were able to dose-dependently inhibit intracellular oxidation induced by 100 mM H_2_O_2_ maintaining an intracellular redox state similar to that of control cells. Fluorescent microscopy results confirming the protective effect of apricot-melanoidins on H_2_O_2_-induced intracellular oxidation are reported in figure 8. A yellow fluorescence pattern, which is the overlapping of the mito-roGFP (green) and MitoTracker Red, is clearly visible in both control and H_2_O_2_-treated cells pretreated with apricot-melanoidins (Fig 8A and D). On the contrary, H_2_O_2_-induced mitochondrial damage is clearly evident in oxidatively stressed cells, which lack the above-mentioned yellow pattern. H2O2-treated cells, have indeed clear mitochondrial damage, and therefore unable to take the Red MitoTracker dye inside (Fig. 8B). As indicated in figure 8C melanoidin alone did not produce intracellular oxidative stress in cultured ECs. Quite similar results are shown concerning ECs expressing the cyto-roGFP (Figure 8E–H), although a clear yellow pattern is not visible due to the expression of roGFP in the cytoplasm. However, a bigger and clearer, punctate red patter is evident in both control and H_2_O_2_-treated cells pretreated with apricot-melanoidins (Fig. 8E and H), as compared with the H2O2-treated ones (Fig. 8F). This is due to the compromised mitochondrial function in H_2_O_2_-treated. No oxidative damage has been produced by melanoidins alone (Fig. 8G).

## Discussion

Oxidative cellular damage during lifetime is emerging as an important factor in the onset and development of many pathological conditions including CVD [22]. Although endogenous antioxidants play an important role in protecting cells against oxidative insults, additional antioxidants (e.g. dietary antioxidants) appear to be required to prevent or to protect living cells from oxidation [1]. In this context, health benefits exerted by plant-derived compounds and extracts have been mainly ascribed to their antioxidant potential and the resulting capability to counteract oxidative-induced damage [13]. However, during food processing and storage, chemical reactions among food components lead to both destruction and formation of phytonutrients [8], therefore whether processed plant foods provide the same benefits as those ascribed to unprocessed ones is uncertain. For instance, melanoidins are heterogeneous polymeric structures formed during food processing in the last stage of the MR whose effects on human health are largely unknown.

Using a previously published procedure [11], [21], [25], fresh apricots were processed by standardized drying and heating conditions, then both fresh and processed fruits were characterized on the basis of commonly recognized parameters namely the variation of color and the browning index, which are both signs of NEBPs formation [27], [29]. As previously reported for prunes [11], the processing-induced elevation of antioxidant capacity was accompanied by a significant increase in the color, as indicated by the decrease of hue angle; suggesting NEBPs may be responsible for the rise in chain-breaking activity observed post- fruit transformation. Indeed, the decrease of hue angle value, which represents the color variation, is an important parameter for the presence of NEBPs and is related to the brown pigment formation caused by NEB reaction during fruit processing [29]. Confirmatory data of NEBPs formation during apricot processing comes from the results obtained after sample blanching. Blanching is a common treatment used to prevent enzymatic browning [30], and therefore variation of color under these experimental conditions can be explained only by the formation of processing-elicited NEBPs. Results depicting the absorbance at 420 nm, which represents the browning index, are further confirmatory data related to NEB due to processing-elicited NEBPs formation [29]. Importantly, these data indicate that NEBPs were presents only in the melanoidins fraction extracted from dried samples. Indeed, as with whole fresh fruit samples, NEBPs were absent on the melanoidins fraction isolated from fresh fruit, confirming both the processing-induced NEBPs formation and the successfull isolation of melanoidin components by the extraction technique employed.

Given the pivotal role played by the endothelium in cardiovascular homeostasis and the involvement of oxidative-induced EC dysfunction in CVD pathogenesis [24], it was reasonable for us to use a human ECs line to investigate the effect of apricot melanoidins on H_2_O_2_-induced oxidative damage. A pathology-associated rise of ROS can trigger mitochondrial membrane permeability promoting the dissipation of MMP and ultimately cell death [31]. Also in our experimental model of oxidative cell death we found a superimposable loss of both MMP and MMA, which clearly indicated the implication of mitochondria in the cell death induced by H_2_O_2_.

In this context, we have previously reported that melanoidins isolated from prunes exert remarkable protection against oxidative-induced ECs death [11], but whether melanoidins from apricots possess the same functional proprieties was so far unknown. Only the partial structure of melanoidins have been elucidated so far and the chemical precursor species responsible remain largely undefined [2], thus melanoidins from different foods may have different structures and activities. In this context, we believe our results may be of importance, since they indicate for the first time that melanoidins isolated from dried apricots, as previously reported from those of prunes [11], exert a significant cellular protection against H_2_O_2_-induced oxidative stress and mitochondrial-mediated EC death. In particular, considering that compounds that participate in melanoidin formation in prunes and apricots differ both analytically and quantitatively, alternate forms of melanoidins may originate from the processing of these two fruits [21], [25], [26].

Fluorescence imaging of ROS in live cells has been widely used to assess oxidative stress in different cellular compartments and under various experimental conditions [32]. However, many of the methods so far employed to determine the levels of intracellular ROS suffer from various pitfalls [32]. A new approach was therefore used in this work to follow H_2_O_2_-induced intracellular oxidation and its possible counteraction by apricot melanoidins. By employing two ECs lines [11], which constitutionally express the redox-sensitive proteins mito- and cyto-roGFP (Fig. 5), we were able to selectively follow changes of the redox state in the cytosolic and mitochondrial compartment. Due to the ability to measure the ratio between the oxidized (GSSG) and reduce form of glutathione (GSH), these two cells lines provided a useful tool for assessing the variation of intracellular redox state, showing significant dynamic range and linear response to increasing doses of the well-known oxidant, H_2_O_2_. Of note, the increased oxidative conditions induced by the applied doses of oxidant were paralleled by a corresponding dose-dependent rise in mitochondrial damage and cellular death, suggesting a relationship between these H2O2-induced phenomena. Under these experimental conditions, apricot melanoidins were able to counteract H_2_O_2_-induced oxidation, maintaining the intracellular redox conditions similar to that of control cells. Consistent with this antioxidant effect is the observed dose-associated protection exerted by apricot melanoidins against the H_2_O_2_-induced mitochondrial impairment and cell death, indicating a tight link between their antioxidant activity and cellular protection.

To our knowledge, this is the first report on the protective effect of apricot melanoidins against oxidative-induced cell death. Moreover, using a novel genetically engineered fluorescence protein to ratiometrically assess the intracellular redox state in living cells, our data confirm and reinforce previously published observations using coffee [9], biscuit [10] and prune melanoidins [11]. Melanoidins indeed, appear to work as antioxidants by positively modulating the GSSG/GSH ratio in favor of the reduced form, and thus favorably preparing the cell to face oxidative insult. In addition, we also detailed the mechanism of cellular protection afforded by melanoidins, which clearly involves protection against intra-mitochondrial oxidation and oxidative-induced mitochondrial impairment assessed as MMA and MMP depolarization. We believe this work adds new insight concerning the effect of processed plant foods on cellular physiology. Indeed, melanoidins from different sources could have different effects, and because of the lack of knowledge in this field, it is imperative that various melanoidins be evaluated under different experimental conditions to determine their effects. Although further studies are required to better characterize the molecular mechanism of melanoidin protection, our findings support the general observation that natural antioxidants from fruits and vegetable can have a cardiovascular protective effect against oxidative stress. To our knowledge, plasma, organs and tissue levels of melanoidins in people are so far unknown, therefore whether the protective effect exerted by melanoidins in cultured cells may be translated in vivo remains to be elucidated. However, there is circumstantial evidence of melanoidin absorption in vivo [33], and consistent with this observation, their antioxidant activity in human volunteers has indeed been reported [34]–[36]. In addition, data obtained with gravimetric techniques allow estimation of a daily intake of about 10 g of melanoidins in a Western diet [33]. These observations indicate that melanoidins may reach in vivo concentrations comparable to the ones we used in vitro, suggesting that our results could be representative of a physiologically relevant in vivo mechanism.

## Materials and Methods

### Chemicals

Unless stated in the text all the reagents used were from Sigma (Sigma, St Louis, MO).

### Sampling, Dehydration and Blanching

The experiments were conducted on the Cafona apricot variety, which has been chosen for its very high content in polyphenols. The fruits were purchased locally at an optimum stage of ripening and those showing defects were discarded. Fruits were size-graded, so that size difference would not affect drying times. Fruits were cut in half along the suture line with a knife and the stone carefully removed by hand. At the end of this procedure, the fruits were immediately checked to eliminate those that had been damaged and then, pre-treated and dried at 75°C as previously described [21]. Before analysis, the dried fruit was packed in co-extruded plastic bags and kept in a freezer −20°C. The blanching was executed as previously reported by boiling the selected samples in water at 90°C for 3 minutes [37].

### Determination of Total Antioxidant Activity

The polyphenol fraction, which was used to assess the antioxidant activity, color variation and browing index, was extracted as previously reported [26]. Antioxidant activity was evaluated using the radical DPPH method as previously described in detail [26] and expressed as-Abs^−3^ min^−1^ g^−1^ of dm.

### Melanoidins Extraction

The extraction of melanoidins was carried out in triplicate, following a previously published method [3], [11], [38]. In detail, 100 g of pitted and ground apricots were defatted with CHCl_3_ while stirring. After solvent evaporation, the operation was repeated twice. Solvent traces were eliminated by rotary evaporation. 200 ml of bi-distilled water were added to this residual solid, and the resulting slurry was sonicated for 30 minutes at 40°C. The water fraction was collected and the operation repeated on the solid phase. The two water fractions were combined and centrifuged at 8400 g for 15 minutes at 15°C, and the supernatant was then evaporated under vacuum at the maximum temperature of 50°C (fraction I). The residual solid was added to that of fraction I and dissolved in 200 ml of ethanol/water (60∶40 V/V), and the resulting slurry was then sonicated for 30 minutes at room temperature. This operation was repeated. The two ethanol:water fractions were combined and centrifuged at 8400 g for 15 minutes at 15°C, and the supernatant was then evaporated under vacuum at the maximum T of 50°C (fraction II). The residual solid was added to that of fraction II and dissolved in 200 mL of 2-propanol/water (50∶50 V/V), and the resulting slurry was then sonicated for 60 minutes at room temperature. This operation was repeated. The two propanol/water fractions were combined and centrifuged at 8400 g C for 15 minutes at 15°, and the supernatant was then evaporated under vacuum at the maximum T of 50°C (fraction III). The remaining solid fraction, which consisted of pieces of fruit, was fraction IV. The yield of each fraction (as g per 100 g of dried fruit) was recorded.

### Determination of Non-enzymatic Browning (NEB)

NEB was assessed by both browing index and color variation. The formation of brown pigment due to the NEB reaction can be estimated as a brown index from spectrophotometric readings at 420 nm [29]. For this reason the selected samples were subjected to a spectrophotometric reading in absorbance mode at 420 nm in a 1 cm glass cuvette (Beckman DU 640 spectrophotometer). The samples were appropriately diluted in water to give absorbance values of <1. These values were used to give an absorbance value per g dm of each diluted fraction. Five measurements were made for each sample. The colorimetric analysis has been carried out as proposed by Mastrocola and Lerici [27]. The peel colour measurement were assessed with a tristimulus colorimeter (Chromameter-2 Reflectance, Minolta, Osaka, Japan), fitted with a CR-300 measuring head. The colour tonality was expressed as L, a*, b* Hunter scale parameters, and “a” and “b” were used to compute hue angle (tan-1 b*/a*) [28], [39]. The measurements have been always done on the same set of 10 fruits selected at the start of the experiment, in order to minimise fruit colour variability.

### Cells Culture Treatments

ECV304 is an endothelial cell line established from the vein of an apparently normal human umbilical cord. This cell line has been proposed as a suitable model for providing novel insights into the mechanisms governing ECs biology under both physiological and pathological conditions [11], [40], [41]. ECV304 were provided by the European Collection of Animal Cell Cultures (ECACC Salisbury, UK). Cells were grown in medium M199 supplemented with 10% fetal bovine serum (Invitrogen, Carlsbad, CA), 100 µg/m1 penicillin, and 100 µg/m1 streptomycin (Invitrogen,). Cells were maintained in a standard culture incubator with humidified air containing 5% CO_2_ at 37°C. The day before each experiment, cells were plated in 24-well plates (Corning, Lowell, MA) at a concentration of 100,000 cells per well and pretreated with melanoidins for 6 hrs before oxidative stress was induced in the last 2 hrs, by treatment with the indicated concentration of hydrogen peroxide (H_2_O_2_). In accord with our previously study using prune melanoidins on human ECs [11], the doses of 2, 6 and 12 µg/ml were tested in our vascular model.

### Cell Viability and Metabolic Assay

Cell viability, for treated and untreated cells, was assessed after 24 hrs by checking the leakage of the cytoplasmatic lactate dehydrogenase (LDH) from cells with a damaged membrane. The amount of LDH released in the medium by death cells was assessed using the kit CytoTox-ONE™ (Promega, Madison, WI). A standard curve with definite amounts of cells (200µ/well) was made, and the release of LDH in the medium was measured after the application of lysis solution (4 µl/well). Plates containing samples were removed from the incubator and equilibrate to 22°C, and then the release of LDH from death cells was measured by supplying lactate, NAD+, and resazurin as substrates in the presence of the enzyme diaphorase. Generation of the fluorescent resorufin product, which is proportional to the amount of LDH, was measured using a GENios plus micro-plate reader (Tecan) with excitation and emission of 560 nm and 590 nm, respectively. By using the standard curve, the amount of LDH release in treated and untreated cell was conversed in number of cells per well. The Mitochondrial Metabolic Activity was assessed as previously reported in 96-well plates (BD Falcon) by using the colorimetric assay MTT (Promega, Madison, WI) [42]. This colorimetric assay measures the reduction of the yellow 3-(4,5-dimethythiazol2-yl)-2,5-diphenyl tetrazolium bromide by mitochondrial succinate dehydrogenase. The yellow tetrazole compound enters the cells and passes into the mitochondria where it is reduced to an insoluble, purple colored, formazan product. The reduction of MTT in isolated cells and tissues is regarded as an indicator of “cell redox activity” [43]. Although evidence exists that the MTT reduction in mammalian cells is also catalyzed by a number of non-mitochondrial enzymes, this reaction is attributed mainly to mitochondrial enzymes and electron carriers [43]–[45]. After treatments cells were added with 20 µl MTT solution (5 mg/ml) in medium M199 and incubated at 37°C in a cell incubator for 60 minutes. At the end of the incubation period, the medium was removed and the cell monolayer was washed twice with HBSS. The converted dye was solubilized with acidic isopropanol (0.04 N HCl in absolute isopropanol), and plates were analyzed at 570 nm using a GENios plus micro-plate reader (Tecan) with background subtraction at 650 nm. Results were expressed as percent of untreated control cells.

### Determination of the Intracellular Redox State

Intracellular redox state was investigated by using the redox-sensing green fluorescent protein (roGFP), which reports the redox state of the GSH/GSSG pool *in vivo* in both plant and mammalian cells [46], [47]. Plasmids coding for roGFP expression were obtained starting from p*CVU55762-roGFP2* (kindly provided by Dr. Andreas J. Meyer, University of Heidelberg, Germany). Cyto-roGFP was obtained by restriction cloning using *Bam*HI and *Not*I restriction enzymes into pcCDNA3 vector (Invitrogen); mito-roGFP2 was obtained by cloning a PCR amplification product into pCMV/myc/mito (Invitrogen) using *Pst*I and *Xho*I sites. Plasmids containing cytoplasmic roGFP2 (cyto-RoGFP) and a mitochondrial targeted roGFP (mit-RoGFP2) were transfected in HCV304 by using the lipofectamine 2000 reagent following the provider protocol (Invitrogen). Transfected cells were selected using 0.8 mg/mL of G418 in the media for 3 to 4 weeks. Positive stably transfectants were selected by serial dilution of G418-resistant clones which constitutively expressed both cyto- and mito-RoGFP2 under a fluorescence microscope (Olympus XI70). RoGFP has two fluorescence excitation maxima at 400 (oxidized form) and 485 nm (reduced form) and display rapid and reversible ratiometric changes in fluorescence in response to changes in ambient redox potential. The ratios of fluorescence from excitation at 400 and 485 nm indicate the extent of oxidation and thus the redox potential while canceling out the amount of indicator and the absolute optical sensitivity [47]. In place of confocal imaging analysis we used a recently developed fluorometer-based method for monitoring roGFP oxidation [11], [48]. Fluorescence measurements were performed in clear 24-well plates (Corning, Lowell, MA) on a fluorescence plate reader GENios plus (Tecan, Männedorf, CH) from the upper side using multiple reads per well (the read pattern was square, and the number of reads was 2×2). Cells were excited by using 400 and 485 nm filters and fluorescence values were measured using 535 nm emission filter. For background correction emission intensities were determined for non-transformed cells (4 discs each experiment) exposed to same excitation wavelengths under the same conditions. These values were averaged and subtracted from the fluorescence values of roGFP2. The degree of oxidation of the roGFP2 was estimated from the ratios of light intensities obtained during 1-min intervals under 400- and 485-nm excitation. Treatment-induced variations of roGFP2 oxidation were estimated by comparison with roGFP oxidation in control untreated cells.

### Measurement of Mitochondrial Membrane Potential

Measurement of mitochondrial membrane potential (MMP) was performed with the JC-1 stain (Invitrogen), a lipophilic cation fluorescent dye that accumulates in mitochondria in a MMP-dependent manner, showing red fluorescent JC-1 aggregates (590 nm emissions) at higher MMP. When MMP decreases, JC-1 aggregates depart from mitochondria and change to green fluorescent JC-1 monomers (535 nm emissions). Therefore, the ratio of the red signal to the green can been used to detect the occurrence of MMP depolarization in the early stages of cell death due to mitochondrial damage [49], [50]. After treatments cells were incubated at room temperature in the dark with 5 µg/ml JC-1 in HBSS for 30 minutes. The cells were then washed twice with HBSS and fluorescence levels were immediately acquired with excitation and emission wavelengths set at 535 and 590 nm, respectively, for red fluorescence, and 485 and 535 nm, respectively, for green fluorescence. Measurements were performed in clear 24-well plates (Corning, Lowell, MA) on a fluorescence plate reader GENios plus (Tecan, Männedorf, CH) from the upper side using multiple reads per well (the read pattern was square, and the number of reads was 2×2). For each sample, the results were calculated as the ratio (red/green) of fluorescence of sample, averaged after the fluorescence values had been corrected for the background and protein content.

### Staining and Fluorescence Visualization

MitoTracker Red CMXRos (Invitrogen, catalog # M7512) is the oxidized form of a dye that can be taken up into the mitochondria of live cells utilizing their uniquely high membrane potential. This dye is retained in the mitochondria after fixation and therefore can be used to label/stain mitochondria followed by additional immunocytochemistry. For the staining, cells have been grown on glass coverslips inserted inside the multi-wells. At the end of experiments, the culture media has been removed and the cells monolayer washed with pre-warmed PBS. Then the pre-warmed solution containing the MitoTracker® probe has been added to each well (final probe concentration of 300 nM) and the cells have been incubated at 37°C for 30 minutes. After the staining was complete, cells were fixed with a solution of 4% formaldehyde in complete growth medium at 37°C for 15 minutes, and the permeabilized for 10 minutes in PBS containing 0.2% Triton® X-100. Hoechst 33342 (SIGMA, catalog# B2261), is part of a family of blue fluorescent dyes commonly used to stain DNA. After fixation/permeabilization, the Hoechst dye has been added to the cells at a final concentration of 0.12 µg/ml. The dye has been left to incubate with the cells for 15 minutes and then the cells monolayer has been washed for five times with PBS before visualization. Fluorescence visualization of fixed cells has been performed on a Olympus BX 51 microscope, using a 20, 40, and 100× objectives with Numerical Aperture (NA) of 0,70, 1,00 and 1,35 respectively.

### Statistical Analysis

Data were expressed as means ± S.E.M. of three or four different experiments. One-way analysis of variance (ANOVA) followed by a post-hoc Newman-Keuls Multiple Comparison Test were used to detect differences of means among treatments with significance defined as P<0.05. Statistical analysis was performed using GraphPad Prism version 5.00 for Windows, GraphPad Software, San Diego California USA.
